# Most-enhancing tumor volume by MRI radiomics predicts recurrence-free survival “early on” in neoadjuvant treatment of breast cancer

**DOI:** 10.1186/s40644-018-0145-9

**Published:** 2018-04-13

**Authors:** Karen Drukker, Hui Li, Natalia Antropova, Alexandra Edwards, John Papaioannou, Maryellen L. Giger

**Affiliations:** Department of Radiology, MC2026, 5841 S Maryland Ave, Chicago, IL USA

**Keywords:** Breast cancer survival, Dynamic contrast-enhanced breast MRI, Most-enhancing tumor volume, Radiomics

## Abstract

**Background:**

The hypothesis of this study was that MRI-based radiomics has the ability to predict recurrence-free survival “early on” in breast cancer neoadjuvant chemotherapy.

**Methods:**

A subset, based on availability, of the ACRIN 6657 dynamic contrast-enhanced MR images was used in which we analyzed images of all women imaged at pre-treatment baseline (141 women: 40 with a recurrence, 101 without) and all those imaged after completion of the first cycle of chemotherapy, i.e., at early treatment (143 women: 37 with a recurrence vs. 105 without). Our method was completely automated apart from manual localization of the approximate tumor center. The most enhancing tumor volume (METV) was automatically calculated for the pre-treatment and early treatment exams. Performance of METV in the task of predicting a recurrence was evaluated using ROC analysis. The association of recurrence-free survival with METV was assessed using a Cox regression model controlling for patient age, race, and hormone receptor status and evaluated by C-statistics. Kaplan-Meier analysis was used to estimate survival functions.

**Results:**

The C-statistics for the association of METV with recurrence-free survival were 0.69 with 95% confidence interval of [0.58; 0.80] at pre-treatment and 0.72 [0.60; 0.84] at early treatment. The hazard ratios calculated from Kaplan-Meier curves were 2.28 [1.08; 4.61], 3.43 [1.83; 6.75], and 4.81 [2.16; 10.72] for the lowest quartile, median quartile, and upper quartile cut-points for METV at early treatment, respectively.

**Conclusion:**

The performance of the automatically-calculated METV rivaled that of a semi-manual model described for the ACRIN 6657 study (published C-statistic 0.72 [0.60; 0.84]), which involved the same dataset but required semi-manual delineation of the functional tumor volume (FTV) and knowledge of the pre-surgical residual cancer burden.

## Background

There is a large variation in the clinical presentation of, and outcome of, breast cancer in women. It has been shown that in many instances biological biomarkers, i.e., features, of the primary tumor correlate with outcome [1–5]. The availability of biomarkers that can be used to assess outcome as early and as accurately as possible is crucial to the development of successful targeted and personalized breast cancer therapies. Methods to assess such biological biomarkers for the prediction of outcome, however, may be invasive, expensive, not repeatable, or not widely available. Our hypothesis is that magnetic resonance (MR) image-based features obtained through quantitative image analysis will prove useful as non-invasive biomarkers for the assessment of, and prediction of, the response of breast cancer to neoadjuvant therapy. The goal of our research is to develop automatic and quantitative image-based surrogate biomarkers of breast cancer tumors for use in predicting recurrence and in association with recurrence-free survival, ultimately aiding in patient management. Moreover, our goal is to base predictions only on data available “early on” during patient treatment, i.e., data available pre-treatment and after the first cycle of neoadjuvant chemotherapy.

In the work presented here, we validated a single image-based biomarker for predicting recurrence and association with recurrence-free survival using MR images of breast tumors from the American College of Radiology Imaging Network (ACRIN) trial 6657, which was a multicenter study of contrast-enhanced MR imaging to assess breast tumor response to neoadjuvant chemotherapy [6]. A previous analysis of the ACRIN 6657 trial by Hylton et al. [7] showed that MR imaging was more strongly associated with pathologic response after neoadjuvant chemotherapy than clinical examination, with the greatest advantage measured early in treatment by using a volumetric measurement of tumor response. Hylton et al. [8] also demonstrated that the functional tumor volume, determined from dynamic contrast-enhanced MR images in a semi-manual manner, was predictive of recurrence-free survival. In this paper, we present our automatic and quantitative radiomics method for the determination of the most enhancing tumor volume as an image-based biomarker in the task of predicting recurrence and association with recurrence-free survival. The method proposed here is completely automated except for the manual localization of a single seed-point in the approximate tumor center. Performance is assessed for our automated method – and compared to that for the functional tumor volume – for the prediction of recurrence, association with recurrence-free survival, and through Kaplan-Meier survival analysis.

## Methods

### Dataset

Dynamic contrast-enhanced magnetic resonance images (DCE-MRI) used in this work were obtained from a de-identified publicly available dataset and hence were IRB exempt for our study. The dataset has been described in detail elsewhere [8]. It was obtained from the ACRIN 6657 study and is available on The Cancer Imaging Archive [9, 10]. Women with breast cancers measuring 3 cm or greater and who were scheduled to receive anthracycline-based neoadjuvant chemotherapy, were eligible for ACRIN 6657, and 237 women were accrued of whom 162 ultimately were enrolled (mean age 49 years, range 27-68 years). Women receiving non-anthracycline chemotherapy agents, such as trastuzumab, were excluded from ACRIN 6657. In our study, we used the images from the baseline exams obtained within 4 weeks before the start of chemotherapy, and those obtained at early treatment, which is defined as at least 2 weeks after the first cycle and before the second cycle of chemotherapy. Not all women (cases) were imaged at all treatment time points, resulting in a different number of exams being available at each treatment time point (Table 1). Of the 162 primary cancers, 78 were hormone-receptor positive (estrogen and/or progesterone receptor positive) and human epidermal growth factor receptor type 2 (HER2) negative, 41 were HER2 positive, 40 were triple negative, and for three the hormone receptor status was unknown.

**Table 1:** Number of exams available (for a total of 162 women) at each treatment time point (with the number of cases with a recurrence vs.no recurrence in parentheses)

	Exams with at least 3 acquisitions^*^	Functional Tumor Volume	Both
Pre-treatment baseline	141 (40 vs. 101)	137 (39 vs. 98)	137 (39 vs. 98)
Early treatment	142 (37 vs. 105)	143 (38 vs. 105)	141 (37 vs. 104)
Both at baseline and early treatment	127 (37 vs. 90)	124 (26 vs. 88)	123 (36 vs. 87)

*For automated tumor segmentation, a dynamic contrast-enhanced MR exam needed at a minimum to consist of a pre-contrast and two post-contrast images; At baseline, all available exams met this criterion while at early treatment a single exam was eliminated from our automated analysis

Within the ACRIN 6657 study, recurrence-free survival was assessed for each patient based on clinical examination and mammography after surgery. The length of recurrence-free survival was defined as the time from initial surgery to local or distant recurrence or the time to last follow-up in patients without evidence of recurrence. We did not discriminate between women with local and distant recurrences, and as endpoint we used the length of recurrence-free survival.

MRIs were obtained using 1.5 T field-strength MR imaging systems [8] and we only used the dynamic contrast-enhanced series (gadolinium-based contrast agent). In-plane spatial resolution was ≤1 mm and slice thickness ≤ 2.5 mm. All but one of the available exams had a single pre-contrast image and two images acquired at about 2 ½ minutes and 7 ½ minutes post contrast-injection, respectively (Table 1).

### Functional tumor volume (FTV)

Apart from the MR images, the functional tumor volume was made available for most exams via the Cancer Imaging Archive (Table 1) [10]. The functional tumor volume (FTV) had been determined in a semi-automated fashion [11] using the signal enhancement ratio method [12]. Their method requires the manual placement of rectangular regions of interest (ROIs) on maximum intensity orthogonal projection images to delineate a 3D rectangular volumetric ROI that completely encloses a given tumor. Moreover, the method requires manual drawing of irregular ROIs to exclude enhancement regions within the 3D ROI that obviously do not correspond to the tumor if those are present (such as the heart or vessels) and manual adjustment of threshold values for the percent early enhancement and signal enhancement ratio, if so desired. Subsequently, the signal enhancement ratio – a measure combining contrast enhancement and washout – and percent early enhancement are calculated for each voxel in the 3D ROI and thresholded to obtain the FTV. For comparison to our methods and results, we used the published values for FTV that were provided with the images from the Cancer Imaging Archive and which were determined with a variable threshold for the percent early enhancement (default 70% but modified on a by-case basis as needed) and zero threshold for the signal enhancement ratio [11].

### Most enhancing tumor volume (METV)

In our method, for the calculation of the most enhancing tumor volume (METV), automated tumor segmentation and radiomics assessment required, at a minimum, the availability of a pre-contrast, first post-contrast, and second post-contrast image within an exam. As mentioned above, one of the exams (at early treatment) failed to meet this criterion (having only a single post-contrast image available) and was not analyzed. After placement, via manual localization, of a single seed-point on the approximate tumor center in only one of the slices within an exam, each tumor was automatically segmented in 3D by a fuzzy c-means technique using the presence of contrast agent within each voxel over time within an automatically determined volume-of-interest centered at the manually-indicated approximate lesion center [13]. This method is well-established and has been used extensively in its original form requiring identification of a region of interest as well as an updated version used in this work requiring only the manual indication of a seed-point [14–20]. In short, fuzzy c-means clustering is an *unsupervised* pattern recognition technique which was used to partition voxels into tumor and non-tumor groups. In order to do so, the kinetic curve was calculated for each voxel from the contrast enhancement over time including all available image acquisitions within a DCE-MRI sequence (both pre- and all post-contrast scans). In other words, this is a 4D segmentation method which for each case uses all 3D MR images within a DCE-MRI sequence. Voxels belonging to the surrounding parenchyma typically demonstrate limited contrast uptake over time while the voxels corresponding to a tumor demonstrate substantial contrast uptake early on in the DCE sequence followed by either a washout of contrast agent, a plateau in contrast uptake, or a continuous contrast uptake depending on tumor pathology. Using the kinetic curves for all voxels surrounding the manually-indicated approximate tumor center as input, fuzzy c-means partitioned these voxels into tumor and non-tumor groups in an *unsupervised* manner, i.e., without any manual determination of, or adjustment of, threshold values (as would be typical for the determination of FTV).

**Fig. 1:**
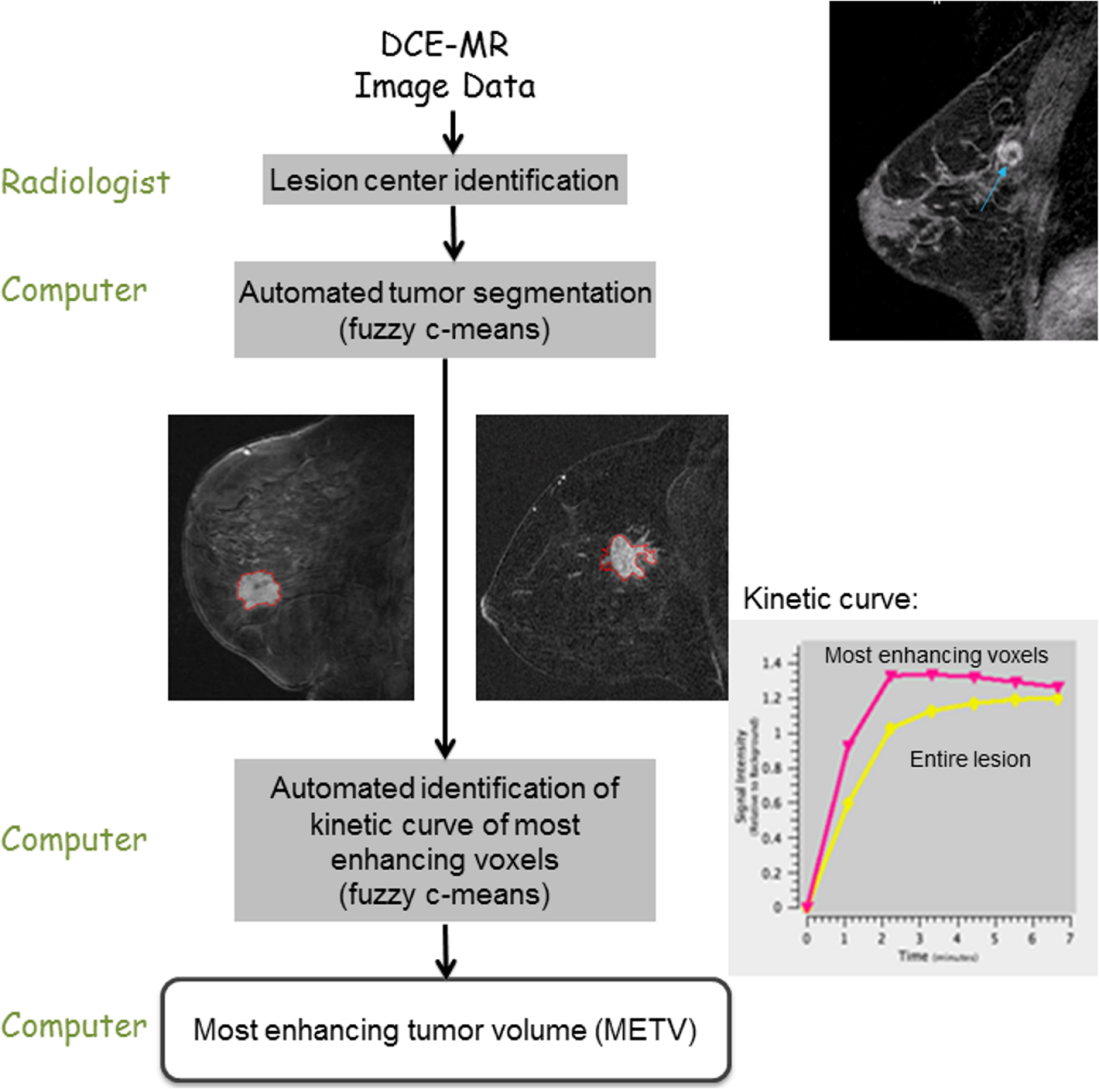
Flowchart of the method for which the only manual input is a seed-point in the approximate lesion center. Example segmentations are shown for a case *without* a recurrence and a small most enhancing tumor volume (left, METV = 114 mm^3^, FTV = 2.8 cm^3^) and a case that developed a recurrence in spite of a small most enhancing tumor volume (right, METV 65 mm^3^, FVT 5.3 cm^3^)

After the automated tumor segmentation, the most enhancing voxels within each segmented tumor were identified through a second fuzzy c-means clustering based on the kinetic curves of only voxels *within* the segmented tumor (Fig. 1) [21]. The voxels that demonstrated the most contrast enhancement within each tumor were identified by fuzzy c-means in an unsupervised manner without any manual determination of, or adjustment of, threshold values. The most enhancing tumor volume (METV) was defined as the aggregate volume of the identified most enhancing voxels. Note that the first fuzzy C-means segmentation used as input an automatically-determined volume-of-interest surrounding each tumor (based on the seed-point location), while the input to the second segmentation step used only voxels within the computer-segmented tumor.

### Performance evaluation and statistical analysis

We examined the values for METV and FTV at the baseline and early treatment exams for patients with and without a future recurrence through the use of box plots. Pearson correlation coefficients [22] were calculated to assess the relationship, if any, between METV and FTV.

For the baseline and early treatment time points, the association of METV and FTV with length of recurrence-free survival (in days) was assessed using a Cox regression model controlling for patient age, race, and hormone receptor status and evaluated by C-statistics [8, 23, 24]. This model was made available by the organizers of the NCI Quantitative Imaging Network (QIN) Breast MRI Metrics of Response (BMMR) challenge.

For the early treatment time point, Kaplan-Meier curves [23, 24] for estimated recurrence-free survival were calculated and compared using a log-rank test using existing Matlab™ code [25]. We compared recurrence-free survival estimates by using METV and FTV cut-points at the lowest (Q1), the median quartile (Q2), and the highest quartile (Q3). Mantel-Haenszel hazard ratios were calculated at the quartile cut-points for METV and for FTV. Non-inferiority was assessed in the task of predicting recurrence-free survival for METV compared to FTV at the early treatment time point by calculating the lower-bound of the one-sided 90% confidence interval for the difference in hazard ratio estimated through bootstrapping (1000 iterations). Kaplan-Meier survival curves were also estimated using the highest quartile cut-point (Q3) for METV at the early treatment time-point by hormone-receptor status subgroup, i.e., for hormone-receptor positive and HER2 negative, HER2 positive, and triple negative subgroups.

## Results

**Fig. 2:**
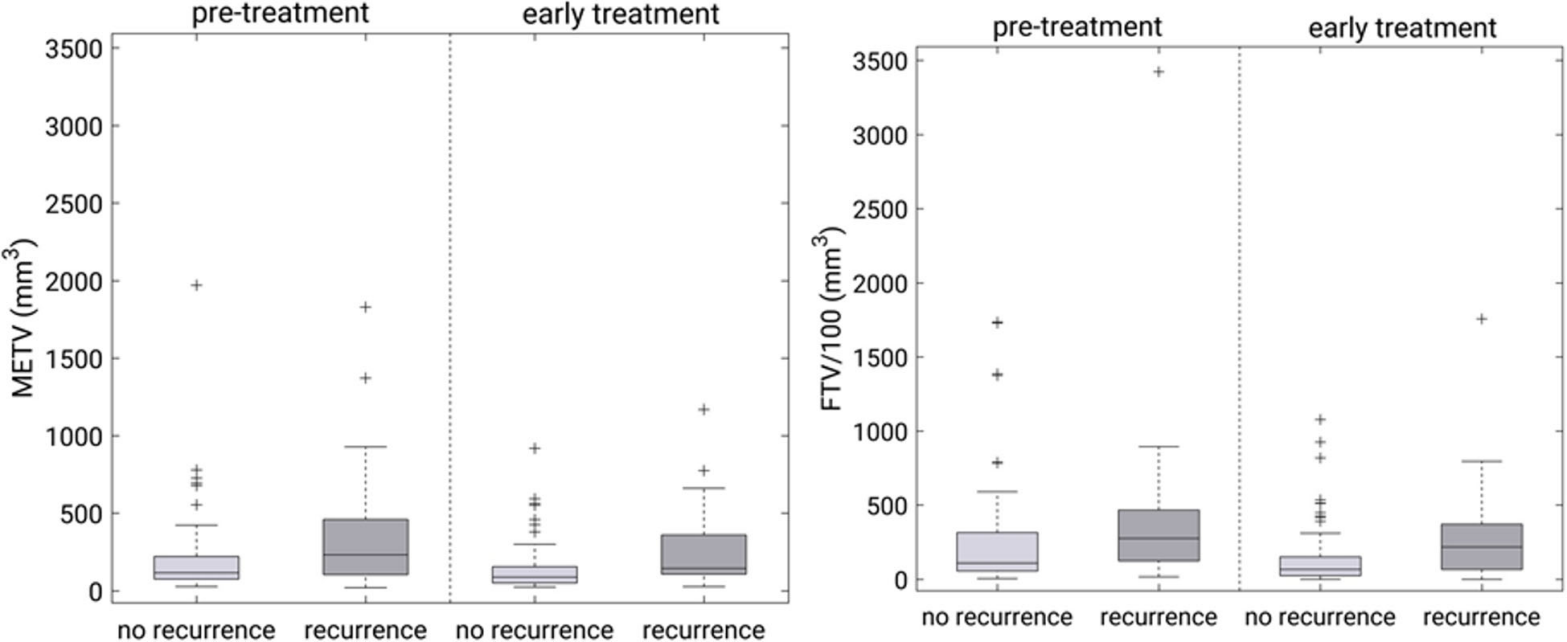
Boxplots of the most enhancing tumor volume, METV (left), and functional tumor volume, FTV (right) [8] at the pre-treatment and early treatment time points for cases without and with a recurrence, respectively. Here, the line within the box marks the median value, the bottom and top of the box mark the 25th and 75th percentile, respectively, the whiskers mark the extremes not considered outliers, and ‘+’ mark individual outliers

METV was generally predictive of future recurrence with a high value indicative of a future recurrence and a low value indicative of the absence of recurrence. The ability of METV to predict a recurrence appeared to be similar to that for FTV [8] at the examined treatment time points (Fig. 2).

The correlation between METV and FTV measurements ranged from slight to substantial [26] when considering all available cases at baseline and early treatment (Table 2). Correlation appeared to be higher at the early treatment time point than at baseline, with correlation coefficients of 0.57 (*p* = 3.6·10^− 12^) and 0.70 (*p* = 4.4·10^− 19^), respectively (Table 2). We found fair correlation between the changes in METV and FTV from baseline to early treatment with a correlation coefficient of 0.29 (*p* = 1.0·10^− 3^).

**Table 2:** Pearson correlation coefficients (with *p*-values) between the most enhancing tumor volume and functional tumor volume for the 123 cases for which both images and functional tumor volumes were available at baseline *and* at early treatment

	All (*N* = 123)	Recurrence (*N* = 36)	No recurrence (*N* = 87)
Pre-treatment baseline	0.57 (3.6·10^−12^)	0.34 (4.0·10^−2^)	0.78 (5.7·10^− 19^)
Early treatment	0.70 (4.4·10^− 19^)	0.69 (3.6·10^− 6^)	0.63 (7.1·10^−11^)
Delta (early treatment-baseline)	0.29 (1.0·10^−3^)	−0.01 (9.5·10^−1^)	0.49 (1.2·10^− 6^)

In the association with length of recurrence-free survival, similar C-statistics were observed for METV and FTV. Both outperformed random guessing with their 95% confidence intervals for the C-statistic excluding 0.5, but the change in METV and FTV over time from pre-treatment to early treatment exams failed to do so (Table 3).

**Table 3:** C-statistic (with 95% confidence interval) for the association of recurrence-free survival (in days) using a Cox regression model controlling for patient age, race, and hormone receptor status

	C-statistic	
	Most enhancing tumor volume METV (automated)	Functional tumor volume FTV (semi-manual) [8]
Pre-treatment baseline	0.69 [0.58; 0.80]	0.67 [0.55;0.79]
Early treatment	0.72 [0.60; 0.84]	0.70 [0.58; 0.82]
Delta (early treatment-baseline)	0.62 [0.50; 0.75]	0.64 [0.50; 0.78]

**Fig. 3:**
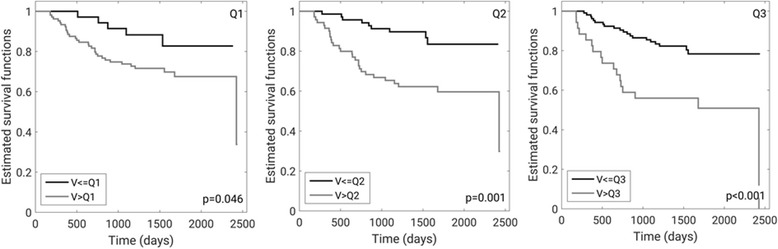
Kaplan-Meier recurrence-free survival estimates for METV at the early treatment time point (*N* = 142 cases) using as cut-points the lowest quartile (Q1, left), median quartile (Q2, middle), and highest quartile (Q3, right) with corresponding *p*-values

Kaplan-Meier plots comparing recurrence-free survival estimates for METV cut-points at the lowest quartile (Q1), the median quartile (Q2), and the highest quartile (Q3) at the early treatment time point all demonstrate statistically significant differences with *p*-values in the log-rank test of 0.046, 0.001, and < 0.001, respectively (Fig. 3). All The hazard ratios corresponding to the shown survival curves for METV (Fig. 3) and the equivalent for FTV demonstrate the potential for the use of either as a decision variable (Table 4). We failed to find any statistically significant differences between survival curves for METV and FTV and the corresponding hazard ratios (all *p*-values> 0.05). The lower bounds of the one-sided 90% confidence intervals for the differences in hazard ratios corresponding to cut points the lowest quartile, the median quartile, and the highest quartile (Table 4) for METV and FTV were − 1.53, − 0.09, and − 6.14, respectively, thus establishing, non-inferiority of METV to FTV only for the median cut point (Q2).

**Table 4:** Hazard ratios at early treatment for Kaplan-Meier curves using cut points at the lowest quartile (Q1), the median quartile (Q2), and the highest quartile (Q3) for METV and FTV (Fig. 3)

	Most enhancing tumor volume METV (automated)	Functional tumor volume FTV (semi-manual) [8]
Lowest quartile Q1	2.28 [1.08; 4.61]	2.68 [1.32; 5.48]
Median quartile Q2	3.43 [1.83; 6.75]	2.23 [1.89; 4.31]
Highest quartile Q3	4.81 [2.16; 10.72]	5.31 [2.59; 12.85]

**Fig. 4:**
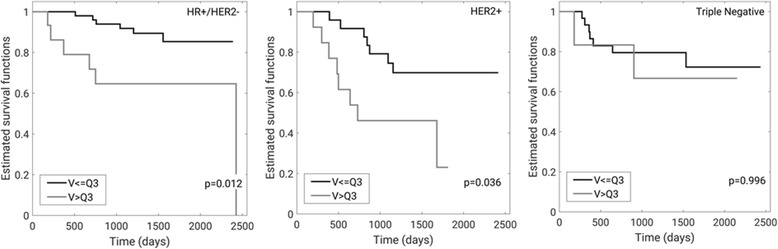
Kaplan-Meier recurrence-free survival estimates for METV at the early treatment time point using the highest quartile cut-point (Q3) with corresponding p-values by hormone-receptor status subgroup: hormone-receptor positive and HER2 negative (*N* = 66, left), HER2 positive (*N* = 38, middle), and triple negative (*N* = 36, right) with corresponding p-values (for 2 cases the hormone receptor status was unknown)

At the early treatment time point (*N* = 140 cases with known hormone receptor status), comparison of the Kaplan-Meier survival curves obtained for the highest quartile (Q3) METV cut-point, demonstrate a statistically significant difference for hormone receptor-positive/HER2 negative (*N* = 66) and HER2 positive sub-groups (*N* = 38) with *p*-values in the log-rank test of 0.012 and 0.036, respectively. For the triple negative cancers (*N* = 36) the comparison fails to demonstrate a statistically significant difference (*p* = 0.996) (Fig. 4).

## Discussion

In this study we showed that the automatically-determined volume of the most enhancing region within a tumor measured on dynamic contrast-enhanced breast MRI, i.e., the most enhancing tumor volume (METV), was predictive of recurrence-free survival for breast cancer patients. Our method is straightforward to use in that it only requires the manual placement (localization) of a seed-point near the approximate tumor center; no bounding box, or variable manual thresholding is used. The method then automatically performs lesion segmentation and feature extraction in real time, making it a promising efficient tool for clinical application. Moreover, our work presented here only involved analysis of the pre-treatment and early treatment (acquired after the first cycle of neoadjuvant chemotherapy) MR exams.

In comparison to the functional tumor volume (FTV) measurements, which were publicly available for the dataset and previously published by Hylton et al. [8], it is interesting to note that the correlation between METV and FTV appeared to be higher at the early treatment time point than at baseline although at both treatment times the overall correlation was substantial [26]. The lower correlation at the pretreatment time point was caused by the low correlation between FTV and METV for patients with a recurrence. Upon closer investigation, however, this low correlation for patients with a recurrence was caused by a single outlier in FTV measurement (an extraordinarily high value for FTV). After removal of this single outlier, the correlation between FTV and METV at the pretreatment baseline was similar to that observed at the early treatment time point (correlation coefficients of 0.69 (*p* = 1.7·10^− 18^) and 0.59 (*p* = 1.8·10^− 4^), for the all patients and those with a recurrence, respectively).

In the work previously published by Hylton et al., using the same dataset [8], the “best” model for predicting recurrence-free survival involved both semi-manual determination of the functional tumor volume (FTV) and knowledge of the pre-surgical residual cancer burden (published C-statistic 0.72, 95% confidence interval [0.60; 0.84]). A direct statistical comparison to that model was not possible but our best performing model including the most enhancing tumor volume at the early treatment exam, patient age, race, and hormone receptor status, achieved a C-statistic of 0.72 [0.60; 0.84]. Note that the residual cancer burden used in the “best” published model is determined post-surgery while our analyses (both for the most enhancing tumor volume and the functional tumor volume) included only information that is already available “early on” in patient treatment.

The performance of the *change* in volume from baseline to after the first cycle of chemotherapy, whether measured through METV or FTV, failed to have any predictive ability in the prediction of recurrence and association with recurrence-free survival. In other words, while both METV and FTV are predictors of recurrence-free survival, their change from baseline to the early treatment time point does not seem to be. This was also observed in the published ACRIN study, where FTV was shown to be a stronger predictor of recurrence –free survival than pathologic complete response [8]. In clinical practice, the change in tumor size is used an as indicator of treatment success. For size measurement, the response evaluation criteria in solid tumors (RECIST) refer to a set of published rules used to assess tumor burden in order to provide an objective assessment of response to therapy. They were initially introduced in 2000 and have undergone subsequent revision in 2009 [27]. In RECIST, for example, partial response is defined as a 30% decrease in the sum of all target lesions in longest axis measurement. So, while the change in tumor size is a clinically-used assessment of “short-term” response to therapy, the *change* in the functional tumor size does not seem to be a strong predictor of “long-term” treatment success in terms of recurrence-free survival [8].

The Kaplan-Meier curves for recurrence-free survival estimated from cut-points of METV at the early treatment time-point were similar to those for FTV published by Hylton et al. [8]. When recurrence-free survival was investigated by hormone receptor status subgroup, however, differences between the two approaches seem to become apparent. While using the highest quartile cut-point for FTV at the early treatment time-point the log-rank test yielded a statistically significant difference *only* for the triple-negative subgroup [8], performing the same analysis for METV yielded statistically significant differences for the hormone-receptor positive/HER2 negative, and HER2 positive subgroups while the difference for the triple negative subgroup failed to reach statistical significance (Fig. 4).

A limitation of this study was the modest size of the dataset. The ACRIN 6657 images and data have only recently become publicly available [9, 10], but the ACRIN 6657 protocol goes back to as far as late 2001. In the years elapsed since then, MR scanners have improved to provide better spatial resolution and better temporal resolution providing more numerous, and more closely-spaced in time, higher quality acquisitions in a dynamic-contrast enhanced MRI sequence. These factors will likely result in improved computerized analysis (for both the automated METV and probably also for the semi-manual FTV). Another concern was that the ACRIN 6657 dataset was acquired before trastuzumab came into routine clinical use (for HER2 positive breast cancers), and that no patient contributing to the dataset was treated with trastuzumab or biosimilars. In order to continue our investigations into the prediction of response to therapy and recurrence-free survival, we are collecting a dataset at our own institution of pre- and post-treatment MR images of women with node-positive locally advanced breast cancers undergoing current clinical treatment protocols with approximately 70 cases collected to date. The ACRIN 6657 image data, however, remains very valuable since it was the result of a multi-year, multi-center clinical trial, which is very difficult to replicate at a single site.

## Conclusions

Breast MR imaging provides prognostic information about tumor response already before any breast cancer treatment and after one cycle of chemotherapy that can potentially help guide treatment. Most enhancing tumor volume (METV), a quantitative radiomics feature calculated automatically in real time after placement of a seed-point on contrast-enhanced MR imaging, predicts recurrence-free survival for patients who receive neoadjuvant chemotherapy for breast cancer. METV predicts recurrence-free survival as early as pretreatment and after one cycle of standard anthracycline-based chemotherapy; in this study, METV measured pretreatment and after one cycle of chemotherapy, had comparable univariable Cstatistics (0.69, 95% confidence interval [0.58; 0.80] and 0.72 [0.60; 0.84], respectively). Performance of METV in the prediction of recurrence-free survival appeared to be comparable to that for the functional tumor volume (FTV), which is determined in a more semi-manual fashion requiring placement of a volumetric region of interest and, if so desired, manual adjustment of the threshold values for the percent early enhancement and signal enhancement ratio and/or manual exclusion of non-lesion regions of enhancement (univariable Cstatistic after one cycle of chemotherapy of 0.72 [0.60; 0.84] for METV versus 0.70 [0.58; 0.82] for FTV, respectively).

In conclusion, the use of our automatic, computer-extracted most enhancing tumor volume shows promise in the effective and efficient prediction of recurrence and in the association with recurrence-free survival. We investigated only the use of MR exams from “early on” in patient breast cancer neoadjuvant chemotherapy treatment, using only MRI exams acquired at the pre-treatment baseline and after the first cycle of chemotherapy, thus potentially enabling changes to therapy well before excision.

## Abbreviations

ACRIN: American College of Radiology Imaging Network
FTV: Functional tumor volume
METV: Most enhancing tumor volume
QIN: Quantitative Imaging Network
RECIST: Response evaluation criteria in solid tumors
ROI: Region of interest
